# Is radial Extracorporeal Shock Wave Therapy (rEWST) combined with supervised exercises (SE) more effective than sham rESWT and SE in patients with subacromial shoulder pain? Study protocol for a double-blind randomised, sham-controlled trial

**DOI:** 10.1186/s12891-015-0712-1

**Published:** 2015-09-11

**Authors:** Elisabeth Kvalvaag, Jens Ivar Brox, Kaia Beck Engebretsen, Helene Lundgaard Søberg, Erik Bautz-Holter, Cecilie Røe

**Affiliations:** Department of Physical Medicine and Rehabilitation, Oslo University Hospital HF, Ullevål, Postboks 4956, Nydalen, 0424 Oslo Norway

## Abstract

**Background:**

Subacromial shoulder pain is a common complaint. Radial Extracorporeal Shock Wave Therapy (rESWT) has being increasingly used to treat calcific and non-calcific tendinosis, although there is no evidence of the effectiveness of rESWT in non-calcific tendinosis of the rotator cuff. A randomised single blind study showed that the short-term effect of supervised exercises (SE) was significantly better than rESWT on subacromial shoulder pain, but both groups improved. In a clinical trial on achilles tendinopathy rESWT improved the effectiveness of treatment with eccentric loading. The objective of this present study is to evaluate if rESWT in addition to SE is more effective in improving shoulder pain and function compared with sham rESWT and SE in patients with subacromial shoulder pain.

**Methods/Design:**

This is a double blind, randomised sham-controlled trial which is performed at the shoulder clinic at the Department of Physical Medicine and Rehabilitation in Oslo University Hospital, Norway. One-hundred-forty-four patients with subacromial shoulder pain lasting at least 3 months, age from 25 to 70 years old are included in the trial. Patients are randomly allocated in 1:1 ratio to receive either rESWT or sham rESWT once a week in addition to SE once a week for the initial 4 weeks. Subsequently SE are provided twice a week for 8 weeks. The primary outcome measure is a change in the Shoulder Pain and Disability Index (SPADI) at 24 weeks follow-up. Secondary outcomes include return to work, pain at rest and on activity, function, and health related quality of life. The patients, the physiotherapist providing the exercise regimen and the outcome assessor are blinded to group assignment. The physiotherapist providing the rESWT is not blinded.

**Discussion:**

Because of the extensive use of rESWT in the treatment of subacromial shoulder pain the results of this trial will be of importance and have impact on clinical practice.

**Trial registration:**

ClinicalTrials.gov NCT01441830

## Background

Shoulder pain is a common complaint, and in Norway about half of the population reports to have at least one episode of shoulder pain annually [1]. The most frequent diagnosis is subacromial pain (impingement syndrome or rotator cuff tendinosis are used synonymously) [2, 3]. The exact structures involved in the development of the pain condition are not clear, but the rotator cuff and the subacromial bursa are possible pain generators [4]. Current studies suggest that central mechanisms are involved [5]. In accordance with the complexity of this pain condition, a Cochrane review that has evaluated the physical tests used to identify subacromial pain have concluded that there is extreme diversity in the performance and interpretation of tests, which hinders synthesis of the evidence and/or clinical applicability [6].

**Fig. 1:**
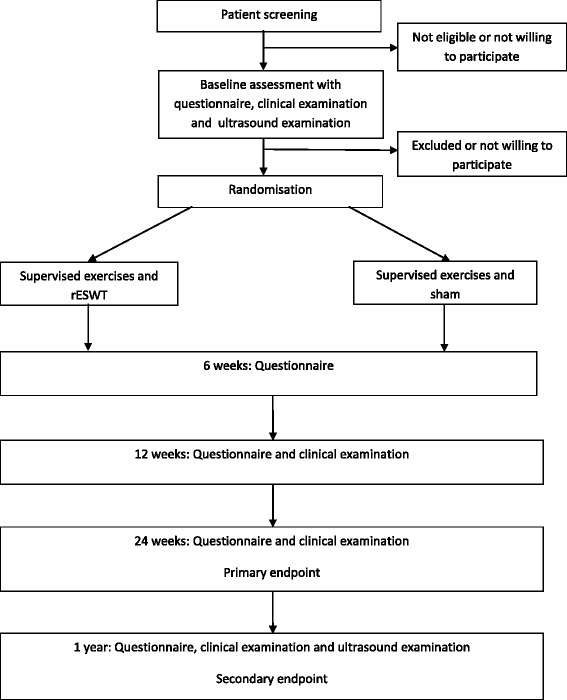
Diagram of enrollment, interventions and follow-up

The patients with subacromial shoulder pain are treated by physicians with different specialities, including general practioners, rheumatologists, doctors in physical medicine and rehabilitation, orthopedic surgeons, and physiotherapists. Many patients with subacromial shoulder pain undergo surgery, even though supervised exercises (SE) have been shown to be as effective as surgery in both short and long term, and better than placebo [7, 8]. The main principles of SE are relearning of “normal” movement patterns which can then be transferred into daily activities. Optimal scapular positioning and centralisation of caput humeri are of importance when performing the exercises before muscle-strengthening program begins [9, 10]. Several studies using similar supervised exercise programs have shown effect on pain and function [11].

Extracorporeal Shock Wave Therapy (ESWT) is another treatment option for patients with subacromial shoulder pain. The proposed mechanisms for the effect of ESWT include pain relief, tissue regeneration and destruction of calcification [12]. A systematic review found level 1 evidence of midterm effectiveness of ESWT in reducing pain and improving shoulder function for patients with chronic calcific tendinopathy of the rotator cuff, but no evidence in favour of ESWT in non-calcific rotator cuff tendinosis [13].

In recent years, a new method of shock wave treatment has been developed: radial Extracorporeal Shock Wave Therapy (rESWT), also called radial pulse therapy (RPT). In contrast to regular focused shock wave therapy (ESWT), rESWT creates a diverging pressure field, which reaches a maximal pressure already at the source, and therefore has a more superficial, but broader, effect than ESWT [12]. This treatment is increasingly used for calcific and non-calcific tendinopathy, probably because it is easier to apply and more affordable than ESWT. For calcific tendinopathy of the shoulder, a systematic review found limited evidence for the benefit of rESWT [13]. However, there is no evidence of the effectiveness of rESWT in non-calcific rotator cuff tendinosis [13, 14].

Musculoskeletal ultrasound might be an important supplement to the clinical examination [4]. Ultrasound can reliably detect calcification, partial and full-thickness tears, bursitis and tendinopathy [15]. Ultrasound examination may be particularly valuable when considering rESWT because rESWT would be expected to be most useful in case of calcification. With respect to routine radiological examination (MRI and ultrasound), a recent study found that structural changes in the rotator cuff and subacromial bursa did not predict short-term outcome after corticosteroid injection therapy [16].

Physiotherapy treatment for subacromial shoulder pain often is a combination of rESWT and exercise therapy. In a recent randomised single blind study the short-term effect of supervised exercises was significantly better than the effect of rESWT on subacromial shoulder pain, but both groups improved [17]. A clinical trial on achilles tendinopathy showed that rESWT significantly improved the effectiveness of eccentric training [18]. However, these studies did not comprise a sham rESWT study arm.

Additionally, previous studies have reported that the prognosis of subacromial shoulder pain is affected by education, work status, polymedication and high baseline pain and disability [16, 19, 20].

## Aims

1. To evaluate whether rESWT in addition to supervised exercises is more effective in improving pain and function (SPADI) compared with supervised exercises and sham rESWT in patients with subacromial shoulder pain at 24 weeks follow-up.
2. To evaluate the influence of demographic and clinical factors on the clinical course of SPADI and sick leave in patients with subacromial shoulder pain during 1-year follow-up.

## Methods

### Study design

This study is a double blind, randomised, sham-controlled trial. All the patients are recruited from the shoulder clinic at the Department of Physical Medicine and Rehabilitation at Oslo University Hospital, Norway.

### Ethics

This study has received approval from the Regional Committee for Medical and Health Research Ethics (2011/255).

### Participants

Patients aged 25–70 years, with subacromial shoulder pain lasting at least 3 months, are eligible for inclusion.

The inclusion criteria are: dysfunction or pain on abduction, normal passive glenohumeral range of motion, pain on at least one of two isometric tests (abduction and/or external rotation) and a positive Hawkins impingement sign [21]. Patients with bilateral shoulder pain are included if both shoulders fulfil the inclusion criteria.

The exclusion criteria are: previous surgery on the affected shoulder, instability, rheumatoid arthritis, full thickness tear of the rotator cuff, cervical radiculopathy, infection, patients considered not being able to fill out questionnaires or follow the treatment, contraindications for shock waves therapy (use of anticoagulant drugs, bleeding disorder, epilepsy, pregnancy or pacemaker), previous experience with shock wave therapy, injection of cortisone in the affected shoulder in the last 6 weeks and SPADI score below 20.

### Randomisation and blinding

The patients who fulfil the inclusion criteria and give their informed consent after having received oral and written information are randomised to one of the two treatment groups: supervised exercises and rESWT, or supervised exercises and sham rESWT. Computer-based block-randomisation with 20 in each block in a 1:1 ratio will be performed. A research assistant not involved in the further management of the patients opens the sealed envelopes and assignes the patients to their respective treatment group. The rESWT and the sham rESWT are subsequently performed by a physiotherapist not involved in any further management of the patients.

The patients, the researchers collecting and analysing the data, the authors and the physiotherapists providing the exercise regimen are all blinded for rESWT/sham rESWT. The blinding will not be revealed until the results are analysed and the interpretation is discussed and written down in two versions, one assuming that treatment A is rESWT, and one assuming that treatment A is sham rESWT.

To evaluate the blinding, all the patients are asked at the 24-week follow up whether they think they have received real rESWT or sham rESWT.

### Interventions

Patients from both intervention groups receive a supervised exercise regimen by experienced physiotherapists. During the initial 4 weeks, they conduct supervised exercises once a week. The last 8 weeks, they perform supervised exercises twice a week. Each supervised exercise session lasts 40 min. In addition, the patients are instructed to conduct home exercises daily.

The patient’s history and functional diagnosis are used as individual guidelines for treatment in the first phase. The main goals of the supervised exercise regimen used in this study are to unload mechanical stress and to normalise dysfunctional neuromuscular movement patterns. Postural exercises, optimal scapular positioning and centralisation of caput humeri are of importance when perfoming the exercises. The physiotherapist supervises and ensures that the patients perform the movements correctly. Then eccentric training, exercises with gradually increasing resistance, and plyometric exercises to improve muscle strength and endurance are performed [9]. Newer research also emphasises a specific and non-generalised treatment approach to this disorder [22]. It is essential to achieve a normal scapulothoracic motion before a muscle-strengthening program can begin, and different studies emphasise the importance of correcting scapular dyskinesis [22–24]. Review articles conclude that the exercise regimen for patients with subacromial shoulder pain are poorly described, but should include postural exercises (posture, shoulder retraction), pendulum exercises for the glenohumeral motion, active assisted AROM, exercises for the rotator cuff, scapular stability training, and stretching/flexibility exercises [11, 25, 26].

The first 4 weeks, the patients receive rESWT or sham once a week in addition to the exercises. The rESWT or sham treatment (SwissDolorClast/EMS) is given by one of two physiotherapists who both went through an application course and training before the study started. The rESWT/sham is given on one to three tendons (supraspinatus, infraspinatus and/or subscapularis), depending on which tendons are painful at isometric tests. Two thousand impulses of shock waves are applied to each painful tendon, with a pressure between 1,5 and 3 bar (depending on what the patient tolerates). We use a power handpiece, which provides an energy of 0,01 – 0,35 mJ/mm2. This handpiece was chosen after advice from the producer (Enimed/SwissDolorClast), physiotherapists with experience in rESWT, and a previous systematic review concluding that future research on rotator cuff tendinosis should focus on high-energy shock wave [27].

The sham rESWT is administered in the exact same way as the rESWT. The sham handpiece is similar to the real handpiece in design, shape and sound, and vibrates exactly like the real handpiece, but no real shock waves are conducted.

Compliance to the treatment is recorded.

### Outcome measurements and assessment

The patients eligible for inclusion are referred to a physician (EK), who examines all the patients at baseline according to a structured protocol including active and passive range of motion, isometric tests, Hawkins sign, examination of the AC joint and biceps tendon. Ultrasound examination of the affected shoulder is performed the same day. The patients also complete a comprehensive standardised questionnaire including primary and secondary outcome measures, the prognostic demographic and clinical factors including sex, age, duration of symptoms, education, drug use, sick leave status and emotional distress. If an MRI has not been performed within the last 3 months, the patients are also referred for an MRI of the affected shoulder.

Self-reported primary and secondary outcome measures are filled in at each follow-up. At 6 weeks after starting the treatment, the patients fill in a short questionnaire. At 12- and 24 weeks and at 1 year they come to follow-up visits where they have an additional clinical examination, all done by the same blinded physician (EK) Fig 1. At 1 year we also perform ultrasound examination.

The primary outcome measure is the Shoulder Pain and Disability Index (SPADI). The SPADI is a self-assessed, validated shoulder score that consists of 13 questions in two subscales, five about pain and eight about function. We use the original version, with each question scoring previous week’s symptoms on a visual analogue scale (VAS). The total SPADI is calculated from averaging the two subscales, and the total score ranges from 0 to 100 with higher score indicating worse pain and disability [28].

Secondary outcomes are return to work, pain at rest and activity measured on an 11-point Likert type scale, function (ability to take something down from a shelf or to carry a 5-kg shoppingbag) measured on an 11-point Likert type scale, and health related quality of life on EuroQol.

EuroQol (EQ-5D and EQ-VAS) is a standardised generic instrument for describing and valuing health related quality of life. The EQ-5D comprises five domains that define health in terms of mobility, self-care, usual activities, pain/discomfort and anxiety/depression. Each domain has three response categories; no problem, some or moderate problems, extreme problems. The resulting health state can therefore be defined as a five-digit number by using the response (1–3) from each dimension. Potentially 243 health states can be defined. The five-digit number is then transformed into a number between -0.56 and 1 with 1 representing the best imaginable health state. The EQ-VAS is a self rating of health status on a vertical VAS from 100 (best imaginable health state) at the top to 0 (worst imaginable health state) at the bottom [29].

The patients are instructed not to attend to any other treatment in the study period. We will register on each follow-up visit if they have received any other treatment since the last visit. The use of analgesics is recorded on baseline and on each follow-up visit.

## Sample size

A previous study indicates an expected standard deviation (SD) of 20 points [17]. To detect a difference in SPADI on 10 points between the groups with a statistical power of 80 % and a significance level of 0.05 we need 50 persons in each group. We have planned to include 144 persons to account for drop out and possibly higher variance.

## Statistical analysis

Descriptive statistics will be used to describe baseline characteristics of the treatment groups. The comparison of between group difference in both primary and secondary outcome variables will be performed according to the intention to treat principle.

Analysis of variance will be applied to evaluate the difference between groups in the change of primary outcome measure (SPADI) between the baseline and 24 weeks follow-up, adjusting for demographic and medical factors such as age, sex, education, sick leave, duration of symptoms, calcification in the rotator cuff, dominant arm affected, bilateral shoulder pain, drug use, emotional distress and compliance to the rESWT treatment. Secondary outcome measures will be assessed with the same approach in separate models. Cox regression will be used to analyse the secondary outcome of return to work at 1 year follow-up.

Finally, mixed model will be applied to explore the variations of the mean SPADI from baseline through the 1-year follow-up within and between patients (between the intervention groups) simultaneously. The effects of demographic and clinical factors on the change of SPADI will also be explored. Number needed to treat will be calculated according to Guyatt et al [30]. Bonferroni correction will be used to correct for multiple testing of secondary outcome.

To deal with missing values of the important analytic variables multiple imputation will be carried out. Estimations from the imputed data will be compared with the estimation from the data with complete values.

## Discussion

Placebo means to please (from latin placere). It is proposed that placebo and opioid analgesics share a neuronal network [31]. The powerful placebo effect is summarized in a recent systematic review [32]. The vertebroplasty trials [33, 34] and the knee arthroscopy trial have demonstrated the effectiveness of sham surgery [35]. One early trial in patients with subacromial shoulder pain reported that sham ESWT was superior to ESWT [36].

Radial ESWT is being increasingly used for musculoskeletal complaints including subacromial shoulder pain, with and without calcification. Current evidence suggests that rESWT may have an effect on calcific tendinopathy of the shoulder, but there is no evidence so far to support the use of rESWT in subacromial shoulder pain without calcification [13, 14, 17, 27]. Most therapists use rESWT on shoulder pain without the use of imaging, and thus do not know whether they are treating a calcific shoulder or not. In this present study we treat patients with subacromial shoulder pain (with and without calcification) with supervised exercises and rESWT. We perform ultrasound examination before treatment and may therefore do subgroup analysis to evaluate if the results are different in the patients with calcific tendinopathy compared to the patients with non-calcific tendinopathy.

In the present study we have included sham treatment to make sure that any difference in results between the groups is due to the rESWT treatment, and not the placebo effect. All the patients get supervised exercises in addition to rESWT or sham, because this is the way most therapists use rESWT today. Exercise therapy is an evidence based treatment option for subacomial sholder pain, both in short- and long term [11, 37].

Because of the abundant use of rESWT, the results of this study will be of major interest. A positive result will support current practice, while no difference between the groups indicates that the use of rESWT for subacromial shoulder pain should not be recommended.
